# Identifying key determinants of cumulative live birth in women with ovarian endometrioma undergoing ethanol sclerotherapy followed by *in vitro* fertilization or intracytoplasmic sperm injection: an interpretable machine learning analysis

**DOI:** 10.3389/fcell.2026.1742816

**Published:** 2026-03-26

**Authors:** Bowen Liu, Yibo Song, Yamei Li, Yudong Liu, Weifen Deng, Yuhua Shi

**Affiliations:** 1 Guangdong Cardiovascular Institute, Guangdong Provincial People’s Hospital, Ganzhou Hospital, Guangdong Academy of Medical Sciences, Guangzhou, China; 2 Guangdong Cardiovascular Institute, Guangdong Provincial People’s Hospital, Guangdong Academy of Medical Sciences, Guangzhou, China; 3 Department of Obstetrics and Gynecology, Center for Reproductive Medicine, Nanfang Hospital, Southern Medical University, Guangzhou, China; 4 Reproductive Medicine Center, Shenzhen Hengsheng Hospital, Shenzhen, China

**Keywords:** cumulativelive birth rate, ethanol sclerotherapy, extreme gradient boosting, *in vitro* fertilization, machine learning, ovarian endometrioma

## Abstract

**Background:**

Ovarian endometriomas impair ovarian reserve and fertility in women of reproductive age. Ethanol sclerotherapy is a fertility-preserving alternative to surgery. Nonetheless, predicting cumulative live birth rates after *in vitro* fertilization remains challenging. This study aimed to develop and validate a machine learning model for predicting the cumulative live birth rate in women with endometriomas who underwent alcohol sclerotherapy followed by assisted reproduction.

**Methods:**

This retrospective cohort study included 194 patients with ovarian endometriomas who underwent ultrasound-guided ethanol sclerotherapy before *in vitro* fertilization or intracytoplasmic sperm injection cycles between January 2020 and December 2024 at our institution. Patients were allocated to the training (135 patients, 70%) and validation (59 patients, 30%) groups. Feature selection used univariate logistic regression (p < 0.10) to identify 19 predictors, which were refined using the Boruta, Recursive Feature Elimination, and maximum relevance minimum redundancy algorithms. Features identified by all methods were selected as the final predictors. Four machine learning algorithms (Decision Tree, Random Forest, Extreme Gradient Boosting, Support Vector Machine) were compared using discrimination, calibration, and utility metrics. SHapley Additive exPlanations analysis was used to interpret the model.

**Results:**

The cumulative live birth rate was 50.0% (97/194). Five predictors were identified: antral follicle count, progesterone level on gonadotropin starting day, downregulation, cyst diameter, and previous live birth history. The Extreme Gradient Boosting model showed optimal performance, with an AUC of 0.830 (95% confidence interval: 0.719–0.941), sensitivity of 0.783, specificity of 0.750, and Brier score of 0.176. SHapley analysis revealed​ that a higher antral follicle count and downregulation positively impacted birth prediction, whereas elevated progesterone levels and larger cyst diameters had negative effects.

**Conclusion:**

We developed an explainable Extreme Gradient Boosting model for predicting cumulative live birth rates in women with ovarian endometriomas after ethanol sclerotherapy and assisted reproductive technology. SHapley Additive exPlanations analysis identified key predictors and revealed their non-linear contributions to outcomes, providing transparent explanations for predictions. This interpretable machine learning approach offers a clinical decision-support tool for patient counseling and treatment optimization, advancing beyond traditional methods in capturing reproductive outcomes.

## 1 Introduction

Endometriosis (EMs) is a chronic, estrogen-dependent inflammatory disease characterized by endometrial-like tissue outside the uterine cavity, affecting 10% of women of reproductive age worldwide (Zondervan et al., 2020). The disease presents with dysmenorrhea, dyspareunia, chronic pelvic pain, and reproductive dysfunction, impacting physical and psychological wellbeing (Marinho et al., 2018; Xu et al., 2025). Ovarian endometriomas (OMAs) are severe manifestations of EMs linked to diminished ovarian reserve, a key determinant of fertility (Daniilidis et al., 2023; Somigliana et al., 2023). This reduction is evidenced by lower anti-Müllerian hormone (AMH) levels and decreased antral follicle count (AFC), which predict ovarian response in assisted reproductive technology (ART) (Practice Committee of the American Society for Reproductive Medicine, 2020). Ovarian reserve depletion occurs due to inflammation, oxidative stress, iron-mediated toxicity and tissue compression (Somigliana et al., 2023). Surgical interventions, especially repeated laparoscopic cystectomy, can further damage ovarian tissue and worsen the decline in ovarian reserve (Muzii et al., 2015).

In response to the recognized iatrogenic risks associated with the surgical management of ovarian endometriomas, ethanol sclerotherapy (EST) has emerged as a promising, minimally invasive, fertility-preserving alternative (Lavadia et al., 2025). This technique uses ultrasound-guided aspiration of endometrioma contents, followed by ethanol instillation into the cyst cavity, inducing chemical ablation while minimizing damage to the surrounding ovarian tissue (Cohen et al., 2017). Lavadia et al. (2025) demonstrated that EST preserves ovarian reserve parameters more effectively than surgical cystectomy, with significantly smaller decreases in AMH and AFC after the intervention. Furthermore, Cohen et al. (2017) reported comparable pregnancy rates between EST and surgical management in their meta-analysis, highlighting the superior ovarian reserve preservation profile of EST. Despite these encouraging findings, EST has limitations, including variable recurrence and incomplete cyst resolution. Heterogeneity in patient selection, protocols, and follow-up has prevented definitive conclusions about optimal EST application.

Among women with OMAs treated with EST prior to *in vitro* fertilization/intracytoplasmic sperm injection (IVF/ICSI), precise prediction of the cumulative live birth rate (CLBR) and the proportion of patients who deliver at least one live infant across all embryo transfers from a single oocyte retrieval cycle remains an important but unresolved clinical challenge (McLernon et al., 2016). CLBR is the most clinically meaningful endpoint in ART, as it captures the complete reproductive potential of a single treatment cycle, encompassing both fresh and freeze-thawed embryo transfers. Predicting CLBR is complex due to multiple interacting factors, including demographics (age, duration of infertility), disease parameters (endometrioma size, laterality, recurrence), ovarian reserve markers (AMH, AFC), controlled ovarian hyperstimulation (COH) protocols, and embryological outcomes (oocyte retrieval, fertilization rate, embryo quality) (Bonavina and Taylor, 2022; Somigliana et al., 2023). Traditional statistical methods, such as logistic regression, frequently fall short of effectively capturing nonlinear relationships and intricate interactions among variables, thereby constraining their predictive accuracy and clinical applicability (Christodoulou et al., 2019).

Machine learning (ML) and artificial intelligence (AI) have transformed reproductive medicine by enabling sophisticated predictive models for high-dimensional data (Orovou et al., 2025; Zhu et al., 2025). ML algorithms, including decision trees (DT), random forests (RF), and Extreme Gradient Boosting (XGBoost), identify complex patterns in large datasets beyond the capabilities of conventional statistical methods (Letterie and Mac Donald, 2020; Bendifallah et al., 2022). ML models have been applied to embryo selection, oocyte competence, ovarian response, and pregnancy outcome prediction (Illingworth et al., 2024; Olawade et al., 2025; Bendifallah et al., 2022). Illingworth et al. (2024) demonstrated a deep learning-based embryo selection-matched manual assessment that reduced observer variability. Similarly, Bendifallah et al. (2022) demonstrated ensemble methods outperformed traditional models. These studies underscore the potential of ML in enhancing clinical decision-making and treatment strategies (Orovou et al., 2025; Zhu et al., 2025; Olawade et al., 2025). However, ML adoption is limited by the “black-box” problem, which lacks transparency (Van Calster et al., 2019). Clinicians need explanations of prediction factors for informed decision making (Ghassemi et al., 2021). SHapley Additive exPlanations (SHAP) effectively addresses this issue by decomposing predictions into interpretable features, thereby bridging the gap between algorithmic sophistication and clinical utility (Lundberg et al., 2020).

To date, no study has developed an explainable machine learning model specifically tailored to predict CLBR in women with OMAs undergoing EST followed by IVF/ICSI, which is a critical gap given the unique reproductive risks posed by both the disease and procedure. Therefore, we aimed to develop and validate an explainable, high-performance model to predict CLBR in this cohort. To achieve this, we implemented a rigorous feature-selection process using multiple machine-learning algorithms to identify robust predictors. We compared the predictive performances of the DT, RF, XGBoost, and Support Vector Machine (SVM) models through comprehensive assessments of discrimination, calibration, and clinical utility. SHAP analysis was used to provide transparent explanations at both the individual and group levels. Ultimately, we sought to deliver a clinically actionable tool to facilitate personalized counseling and support evidence-based treatment decision-making.

## 2 Materials and methods

### 2.1 Study design and ethical approval

This retrospective cohort study was conducted at the Reproductive Medicine Center of Shenzhen Hengsheng Hospital between January 2020 and December 2024. The study protocol was reviewed and approved by the Institutional Review Board and Ethics Committee (HSYY 2022-12–22). Given the retrospective nature of the study and the use of de-identified data, the requirement for informed consent was waived in accordance with the institutional guidelines and the Declaration of Helsinki. All procedures were performed in compliance with the relevant ethical regulations and data protection laws. All analyses adhered to the Transparent Reporting of a Multivariable Prediction Model for Individual Prognosis or Diagnosis (TRIPOD) guidelines to ensure methodological rigor and reproducibility (Collins et al., 2015).

### 2.2 Patient population and selection criteria

#### 2.2.1 Inclusion criteria

Eligibility for inclusion in the study was determined based on the following criteria: (1) female participants aged between 20 and 45 years at the time of ultrasound-guided EST; (2) a diagnosis of OMAs confirmed via transvaginal ultrasound, characterized by unilocular or multilocular cysts with homogeneous, low-level “ground-glass” echogenicity, with at least one cyst measuring 4 cm or larger in diameter; (3) receipt of EST as the primary treatment for OMAs, followed by the initiation of at least one complete IVF/ICSI cycle within 6 months; and (4) availability of comprehensive clinical data, including complete baseline characteristics, COH parameters, embryological outcomes, and follow-up records until the determination of pregnancy outcomes or cycle cancellation.

#### 2.2.2 Exclusion criteria

Patients were excluded from the study if they met any of the following criteria: (1) absence of more than 20% of key predictor variables; (2) history of ovarian surgery for OMAs or adnexal pathologies that compromise ovarian reserve; (3) severe male factor infertility; (4) known chromosomal abnormalities or genetic disorders in either partner; (5) concurrent malignancy or severe systemic disease; and (6) inability to follow up before the determination of pregnancy outcomes.

### 2.3 Intervention

#### 2.3.1 Ultrasound-guided EST

All patients underwent ultrasound-guided EST as the primary treatment for OMAs. The procedure was performed under conscious sedation or general anesthesia, based on clinical indications. Under continuous transvaginal ultrasound guidance, a 17G single-lumen needle was used to aspirate the cyst contents (recorded volume) and saline irrigation was performed. Ninety-five percent ethanol, comprising 50%–80% of the aspirated volume and up to 8 mL, was instilled, retained for 10–15 min, and then re-aspirated. Patients were monitored for complications and discharged on the same or following day. Ultrasound follow-ups at 1 and 3 months were conducted to assess cyst resolution and recurrence (Lavadia et al., 2025; Cohen et al., 2017).

#### 2.3.2 IVF/ICSI treatment procedure

Following EST, patients underwent IVF/ICSI within 6 months. COH protocols were individualized based on age, ovarian reserve, and prior responses (Zhang et al., 2020). Ovarian response was monitored using serial transvaginal ultrasonography and serum sex hormone measurements. When at least two follicles reached 18 mm, final oocyte maturation was triggered by human chorionic gonadotropin (hCG) or a gonadotropin-releasing hormone (GnRH) agonist. Oocyte retrieval under ultrasound guidance was performed 34–36 h later. Oocytes were fertilized using conventional IVF, ICSI, or rescue ICSI, according to the sperm parameters. Embryos were cultured in sequential media under standard conditions, and their quality was assessed using consensus morphological criteria. Embryo transfer was performed on day 3 or 5, based on the development and institutional protocols. Preimplantation genetic testing for aneuploidy (PGT-A) was not routinely performed for the embryos transferred in this study cohort, as it was only recommended for patients with specific clinical indications such as advanced maternal age or recurrent implantation failure, consistent with our center’s standard clinical practice. Progesterone was administered as luteal-phase support. Surplus good-quality embryos were vitrified for subsequent freeze-thaw embryo transfer (FET) cycles.

### 2.4 Data collection and variable definition

#### 2.4.1 Data extraction and quality assurance

All data were extracted from the electronic medical record system (EMRS) by two independent researchers (LBW and LYM). To ensure the accuracy of the data extraction, a consistency check was performed on a randomly selected 20% of the sample. By calculating inter-rater reliability (IRR) metrics, we confirmed the high reliability of our data. For categorical variables, the average Cohen’s Kappa coefficient was 0.902, indicating almost perfect agreement. For continuous variables, the average Intraclass Correlation Coefficient (ICC) was 0.992, indicating good agreement. This rigorous process ensured the accuracy of the final dataset used for analysis. We believe this clarification demonstrates that we took rigorous steps to minimize measurement bias and ensure the high quality of the data used in our analysis.

#### 2.4.2 Baseline and clinical variables

Baseline demographic variables included female age at EST (years), body mass index (BMI), duration of infertility (years), type of infertility (primary or secondary), and previous live birth history (yes/no). The clinical variables included endometrioma characteristics, such as cyst number (single/multiple), cyst laterality (unilateral/bilateral), cyst status (primary/recurrent), and maximum cyst diameter (cm). Ovarian reserve markers were assessed using AMH (ng/mL) and AFC. Detailed parameters of the subsequent IVF/ICSI cycle were documented, including the COH protocol, use of downregulation (yes/no), progesterone on gonadotropin starting day, and number of large follicles (≥18 mm) on hCG day, total gonadotropin dose (IU), duration of stimulation (days), and fertilization method (conventional IVF, ICSI, or rescue-ICSI). The recorded embryological variables included the number of oocytes retrieved, number of mature (MII) oocytes, number of usable embryos, number of good quality embryos, normal fertilization rate, cleavage rate, and blastocyst formation rate.

#### 2.4.3 Outcome measures

The primary endpoint was CLBR, defined as live birth (≥24 gestational weeks) following all embryo transfers (fresh and freeze-thawed) derived from a single oocyte retrieval cycle, as verified by medical records and follow-up (Neves et al., 2023).

### 2.5 Data preprocessing and quality control

#### 2.5.1 Outlier detection and management

For all continuous variables, we performed outlier detection using the standard 1.5×interquartile range (IQR) criterion, where values below Q_1_-1.5*IQR or above Q_3_+1.5*IQR were flagged as outliers. Among the 6 continuous variables, 39 outliers were identified (3.54% of total observations) (Supplementary Figure S1). This low rate suggests that the overall data quality is good.

#### 2.5.2 Missing data handling and imputation

In our dataset, a small proportion of missing values was present. Variables with more than 20% missing values were excluded from the analysis. We assessed the pattern of missing data through visual analysis, where a heatmap showed a scattered, non-systematic distribution of missing values across samples (Supplementary Figure S2). This supported the assumption that the data were Missing At Random (MAR). Consequently, we employed the Multiple Imputation by Chained Equations (MICE) method to handle the missing values. The bar chart illustrating the proportion of missing values across variables is presented in Supplementary Figure S3. In line with current statistical guidelines, we set the number of imputations to m = 10 to ensure the stability of parameter estimates and the reliability of our results.

#### 2.5.3 Normality assessment and transformation strategy

We assessed the normality of all continuous variables. Although some variables exhibited non-normal distributions, we ultimately decided to use the original, untransformed variables for model training. This decision was based on two primary considerations. First, the core machine learning algorithms employed in our study, particularly tree-based models (e.g., RF, XGBoost and SVM), are inherently insensitive to monotonic transformations of features and thus do not strictly require input variables to follow a normal distribution. Second, while we explored methods such as log-transformation, we found that these transformations did not yield significant improvements in the final model performance. Therefore, to maintain the intuitive and straightforward interpretability of the model, especially in the SHAP analysis where original scales are more meaningful, we opted to proceed with the variables on their original scale.

#### 2.5.4 Data spilting strategy

The entire dataset (n = 194) was randomly partitioned into a training set (n = 135) and a validation set (n = 59) at a 70:30 ratio. To ensure the reproducibility of the split, a fixed random seed (random_state = 42) was used. Furthermore, stratified sampling was employed based on the primary outcome to maintain a consistent distribution of outcome events between the two sets.

### 2.6 Feature selection strategy

A rigorous two-stage feature selection process was implemented to identify the most robust and informative predictors of CLBR. In the first stage, univariate logistic regression analysis was performed on the training set for each candidate predictor. Variables with a p-value less than 0.10 were considered potentially relevant and advanced to the second stage. This liberal threshold was deliberately chosen to avoid the premature exclusion of potentially important predictors that might exhibit significance only in multivariable contexts. In the second stage, three complementary feature selection algorithms were applied: (1) the Boruta algorithm, a wrapper method based on random forest that identifies relevant features by comparing their importance with randomly permuted shadow features, run with 100 iterations; (2) Recursive Feature Elimination (RFE), a backward selection method implemented with 5-fold cross-validation using random forest as the base estimator, determining the optimal number of features by the peak cross-validation score; and (3) maximum relevance and minimum redundancy (mRMR), an information-theoretic approach that selects features with maximum relevance to the target while minimizing inter-feature redundancy. To ensure robustness and minimize the risk of overfitting, the final set of predictors was determined by selecting features identified by all three algorithms, representing a consensus approach that retained only the most stable and reproducible predictors across diverse selection methods. To assess for multicollinearity among the five predictors included in the final model, we calculated the Variance Inflation Factor (VIF). The results showed that all variables had VIF values well below 2.0 (range: 1.03–1.26), indicating that multicolli nearity was minimal and not a concern for the stability or interpretation of the model. The detailed VIF values are provided in Supplementary Table S1.

### 2.7 Machine learning model development and performance evaluation

#### 2.7.1 Algorithm selection and theoretical foundation

Four machine learning algorithms representing diverse modeling paradigms were selected for comparative evaluation based on their established performance in clinical prediction tasks and complementary strengths in handling nonlinear relationships and complex interactions.

DT is a non-parametric supervised learning algorithm that recursively partition the feature space into homogeneous subgroups based on feature values that maximize information gain or minimize impurity. The tree structure provides inherent interpretability, with each internal node representing a decision rule and each leaf node representing the predicted outcome. However, single-decision trees are prone to overfitting and exhibit high variance.

RF is an ensemble learning method that constructs multiple decision trees using bootstrap aggregating (bagging) and random feature subsampling and then aggregates predictions through majority voting for classification tasks. By averaging predictions across diverse trees trained on different data subsets and feature combinations, RF reduces variance and improves generalization compared to single DT while maintaining resistance to overfitting.

XGBoost is an advanced implementation of gradient boosting that builds an additive ensemble of weak learners (typically shallow decision trees) sequentially, with each subsequent tree trained to correct the residual errors of the preceding ensemble. XGBoost incorporates regularization terms (L1 and L2) to penalize model complexity, prevent overfitting, and employ second-order gradient information for more accurate optimization. Additional features include column and row subsampling, parallel processing and handling of missing values.

SVM is a kernel-based supervised learning algorithm that constructs an optimal separating hyperplane in a high-dimensional feature space by maximizing the margin between the classes. A radial basis function kernel was employed to capture the nonlinear relationships by implicitly mapping the input features to an infinite-dimensional space.

#### 2.7.2 Hyperparameter optimization and model training

All algorithm hyperparameters were optimized using 5-fold stratified cross-validation with a grid search on the training dataset. We maximized the area under the receiver operating characteristic curve (AUC-ROC), which quantifies the discrimination ability across all classification thresholds. Stratified sampling maintained outcome balance within each fold to mitigate class imbalance bias. The models were then retrained on the complete training dataset using the optimal hyperparameters (those yielding the highest mean cross-validation AUC). The class weights were adjusted using a balanced weighting approach that applies higher penalties to minority class misclassifications.

#### 2.7.3 Model performance evaluation

The model performance was evaluated using training and independent validation sets with complementary metrics for discrimination, calibration, and clinical utility. Discrimination was assessed using the AUC-ROC, where a value of 0.5 indicates no discrimination and 1.0 signifies perfect discrimination. Additionally, threshold-based metrics such as sensitivity, specificity, positive predictive value (PPV), negative predictive value (NPV), F1-score (the harmonic mean of precision and recall), and balanced accuracy (the average of sensitivity and specificity) were calculated at the optimal threshold determined by Youden’s index (sensitivity + specificity - 1). Calibration was assessed using calibration curves across risk deciles, and the Brier score was used as an integrated measure of accuracy, ranging from 0 to 1, where lower values were better. The clinical utility was evaluated using decision curve analysis, which compares the net benefit of the model across threshold probabilities to treat-all and treat-none strategies, with utility supported when the curve of the model lies above both reference lines.

### 2.8 SHAP interpretability analysis

To make the model easier to understand and useful for doctors, we used SHAP analysis on the best model. SHAP, which is based on game theory, shows the extent to which each feature affects predictions by calculating the Shapley values. These values represent the average impacts of each feature. We created SHAP summary plots to rank the features by their importance and show how they affect the predictions using colors. For important features, we created plots to show how the feature values relate to the predictions, including any interactions between them. We also created patient-specific plots to break down the predictions into feature contributions, which helped with personalized risk assessment and clinical advice.

### 2.9 Sample size calculation and statistical analysis

#### 2.9.1 Sample size calculation

The sample size for this machine learning model development study was determined using multiple established criteria for the clinical prediction models. First, we applied the Events Per Variable (EPV) criterion, which is widely used in clinical prediction research. With 97 cumulative live birth events and five final predictor variables, our study achieved an EPV of 19.4, which exceeds the traditional minimum threshold of 10 and approaches the recommended optimal threshold of 20 for robust model development. For machine learning-specific considerations, we referenced the Sample Size Analysis for Machine Learning (SSAML) methodology (Goldenholz et al., 2023), which accounts for the unique characteristics of ML algorithms. With our balanced outcome distribution (50% event rate) and moderate model complexity, the estimated minimum sample size is between 150 and 180 participants, which our study meets. While recognizing that larger sample sizes would further bolster model robustness and generalizability, our calculations indicate that a sample of 194 participants with 97 events offers sufficient statistical power for developing and internally validating machine learning models with predictor variables, especially given the balanced nature of our outcome variable.

#### 2.9.2 Statistical analysis

Statistical analyses were conducted using R (v4.2.2). Continuous variables are presented as mean ± standard deviation for normally distributed data or as median and IQR for non-normally distributed data. Categorical variables are expressed as frequencies and percentages. Group comparisons were performed using t-tests/Mann–Whitney U tests for continuous variables and Chi-square or Fisher’s exact tests for categorical variables. ML models were developed using caret for model training and hyperparameter tuning, with specific implementations through rpart (DT), randomForest (RF), xgboost (XGBoost), and e1071 (SVM). Optimization employed 5-fold stratified cross-validation with a grid search to maximize AUC-ROC. Performance evaluation was performed using pROC (ROC analysis) and caret (classification metrics). The calibration assessment involved rms (calibration curves), the Hosmer-Lemeshow test, and DescTools (Brier score). The clinical utility was evaluated using decision curve analysis). Model interpretability was addressed using shapviz and fastshap for the SHAP analysis, generating summary, dependence, and force plots. To investigate some of the unexpected associations observed in our initial analysis (e.g., lower AFC in the live birth group and higher CLBR in the absence of downregulation), we conducted subgroup analyses. A two-sided p < 0.05 was considered significant.

## 3 Results

### 3.1 Baseline characteristics of enrolled patients

**FIGURE 1:**
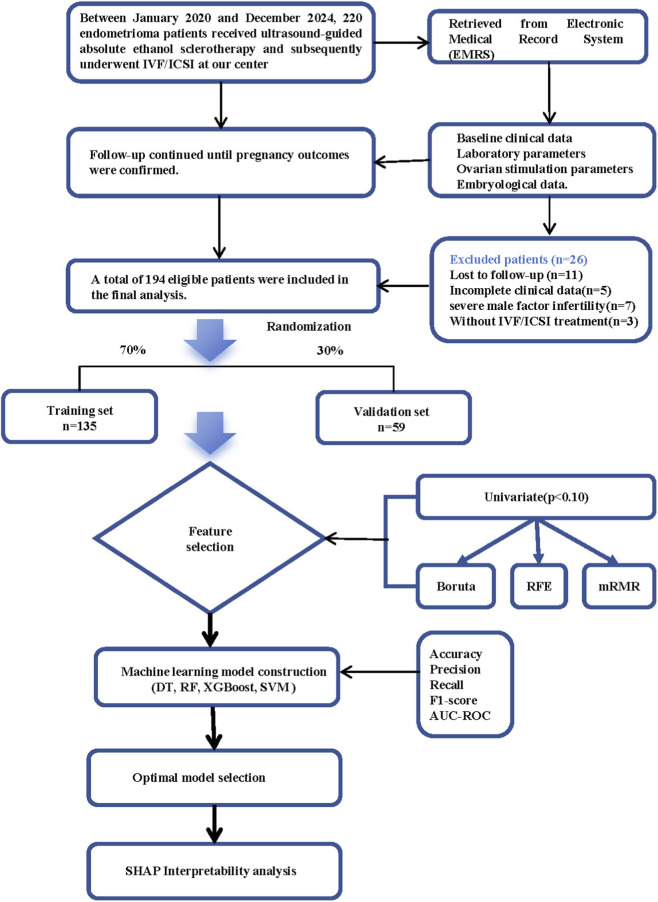
Flowchart of the Study Design and Machine Learning Model Development Workflow. Note: IVF/ICSI, *in vitro* fertilization/intracytoplasmic sperm injection; EMRS, Electronic Medical Record System; DT, Decision Tree; RF, Random Forest; XGBoost, Extreme Gradient Boosting; SVM, Support Vector Machine; RFE, Recursive Feature Elimination; mRMR, max-relevance and min-redundancy; AUC-ROC, area under the receiver operating characteristic curve; SHAP, SHapley Additive exPlanations.

Medical records of 220 patients were collected. Based on the set rules, 194 patients were included in the study. The patients were divided into two cohorts: 135 individuals were assigned to the training group, while 59 individuals were allocated to the validation group, adhering to a 7:3 ratio (Figure 1). We compared the baseline characteristics of the training and validation groups. Significant differences between the training and validation groups were observed in basal testosterone levels (28.22 [19.44; 34.56] vs. 22.75 [18.43; 28.23] ng/dL, p = 0.045) and AFC (11.00 [6.00; 16.00] vs. 9.00 [5.00; 13.00], p = 0.030). Nevertheless, the overall similarity across most parameters supports the validity of the dataset partitioning for model training and validation (Table 1). The CLBR was 50.0% (97/194). Patients in the live birth group were significantly younger (32.00 [30.00; 34.00] vs. 34.00 [31.00; 37.00] years, p < 0.001), had a lower BMI (20.55 [18.75; 22.66] vs. 21.63 [19.49; 23.44] kg/m^2^, p = 0.042), shorter infertility duration (2.00 [1.00; 4.00] vs. 4.00 [2.00; 6.00] years, p = 0.004), and higher AMH levels (3.08 [1.84; 4.65] vs. 2.53 [1.26; 3.71] ng/mL, p = 0.013) than those in the non-live birth group. Interestingly, the live birth group demonstrated a significantly lower AFC (9.00 [5.00; 13.00] vs. 13.00 [7.00; 17.00], p < 0.001), while no significant differences were observed in endometrioma characteristics, including cyst diameter, laterality, and recurrence status, between the two groups (Table 1). Furthermore, the Live Birth group yielded superior embryological outcomes during the IVF cycle, including a higher number of oocytes retrieved, MII oocytes, and available embryos (p < 0.05) (Supplementary Table S2).

**TABLE 1:** Baseline characteristics of study population.

Variables	All participants (n = 194)	Live birth group (n = 97)	Non-live birth group (n = 97)	p^*^	Training set (n = 135)	Validation set (n = 59)	p^#^
Female age (years)	33.00 [30.00; 35.75]	32.00 [30.00; 34.00]	34.00 [31.00; 37.00]	<0.001	33.00 [30.00; 35.00]	33.00 [31.00; 36.00]	0.585
Male age (years)	34.00 [32.00; 37.00]	34.00 [31.00; 36.00]	35.00 [32.00; 38.00]	0.018	34.00 [32.00; 36.50]	35.00 [31.00; 38.00]	0.524
BMI (kg/m^2^)	21.20 [18.93; 23.12]	20.55 [18.75; 22.66]	21.63 [19.49; 23.44]	0.042	21.22 [18.98; 23.19]	21.09 [18.95; 22.62]	0.939
Duration of infertility (years)	3.00 [2.00; 5.00]	2.00 [1.00; 4.00]	4.00 [2.00; 6.00]	0.004	3.00 [2.00; 5.00]	3.00 [2.00; 5.00]	0.683
bFSH (mIU/mL)	6.44 [5.29; 7.65]	6.40 [5.42; 7.48]	6.64 [5.15; 7.85]	0.801	6.41 [5.29; 7.60]	6.51 [5.27; 7.95]	0.942
bLH (mIU/mL)	3.59 [2.77; 4.90]	3.59 [2.93; 4.95]	3.59 [2.70; 4.83]	0.312	3.59 [2.71; 4.94]	3.70 [3.00; 4.68]	0.600
bPRL (ng/mL)	13.05 [7.89; 17.79]	13.25 [3.23; 18.28]	12.99 [9.15; 16.85]	0.489	12.90 [7.90; 17.93]	14.02 [8.38; 17.02]	0.777
bE_2_ (pg/mL)	137.67 [87.89; 191.14]	122.03 [74.47; 174.61]	145.50 [94.09; 194.80]	0.063	137.81 [88.41; 190.64]	137.53 [88.17; 199.62]	0.745
bT (ng/dL)	25.77 [19.30; 33.98]	25.34 [19.30; 32.26]	25.92 [18.72; 33.98]	0.733	28.22 [19.44; 34.56]	22.75 [18.43; 28.23]	0.045
bP (ng/mL)	0.19 [0.11; 0.31]	0.19 [0.12; 0.29]	0.22 [0.09; 0.31]	0.762	0.19 [0.11; 0.30]	0.19 [0.11; 0.30]	0.695
AMH (ng/mL)	2.83 [1.56; 4.02]	3.08 [1.84; 4.65]	2.53 [1.26; 3.71]	0.013	2.67 [1.46; 4.07]	3.06 [1.95; 3.99]	0.461
AFC	10.00 [6.00; 15.00]	9.00 [5.00; 13.00]	13.00 [7.00; 17.00]	<0.001	11.00 [6.00; 16.00]	9.00 [5.00; 13.00]	0.030
Cyst diameter (cm)	5.54 [4.83; 7.46]	5.58 [4.78; 7.34]	5.48 [4.86; 7.51]	0.965	5.50 [4.84; 7.38]	5.56 [4.82; 7.59]	0.994
Cyst number	​	​	​	0.090	​	​	0.905
Single	132 (68.04%)	60 (61.86%)	72 (74.23%)	​	91 (67.41%)	41 (69.49%)	​
Multiple	62 (31.96%)	37 (38.14%)	25 (25.77%)	​	44 (32.59%)	18 (30.51%)	​
Cyst laterality	​	​	​	0.859	​	​	0.368
Unilateral	154 (79.38%)	78 (80.41%)	76 (78.35%)	​	110 (81.48%)	44 (74.58%)	​
Bilateral	40 (20.62%)	19 (19.59%)	21 (21.65%)	​	25 (18.52%)	15 (25.42%)	​
Cyst status	​	876	​	0.594	​	​	0.897
Primary	154 (79.38%)	75 (77.32%)	79 (81.44%)	​	108 (80.00%)	46 (77.97%)	​
Recurrent	40 (20.62%)	22 (22.68%)	18 (18.56%)	​	27 (20.00%)	13 (22.03%)	​

Data are presented as mean ± standard deviation (SD) for normally distributed continuous variables, median [interquartile range (IQR)] for non-normally distributed continuous variables, and n (%) for categorical and count data. p^*^ compares the live-birth and non-live-birth groups; p^#^ compares the training and validation sets. A two-sided p < 0.05 was considered statistically significant. BMI, body mass index; bFSH, basal follicle-stimulating hormone; bLH, basal luteinizing hormone; bPRL, basal prolactin; bE_2_, basal estradiol; bT, basal testosterone; bP, basal progesterone; AMH, anti-Müllerian hormone; AFC, antral follicle count.

### 3.2 Feature selection for the predictive model

**FIGURE 2:**
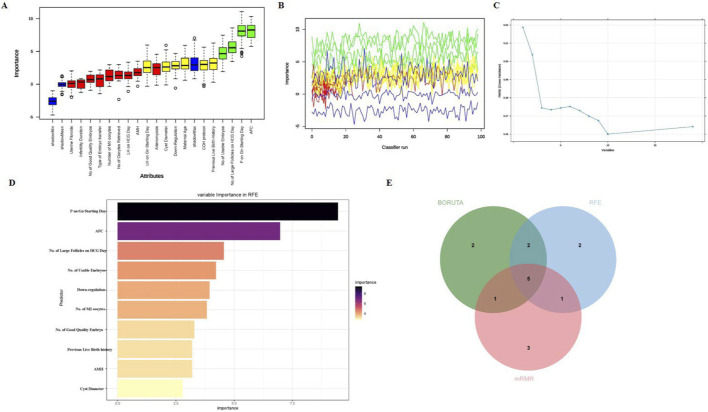
Feature Selection Using Three Complementary Algorithms. **(A)** Boruta feature importance ranking color-coded: blue = rejected, yellow = tentative, red = confirmed, green = critical. **(B)** Boruta iterative process across 100 runs, showing feature importance trajectories. **(C)** RFE cross-validation curve identifying the optimal feature number. **(D)** RFE variable importance ranking plot. **(E)** Venn diagram showing intersection of features selected by Boruta, RFE, and mRMR. Note: AFC, antral follicle count; HCG, human chorionic gonadotropin; AMH, anti-Müllerian hormone; M2 oocytes, metaphase II oocytes; Pn, pronucleus; RFE, Recursive Feature Elimination; mRMR, max-relevance and min-redundancy.

To construct an accurate predictive model, we first conducted a systematic feature-selection process. In the initial step, we performed a univariate logistic regression analysis on all potential predictors in the training group. By applying a p-value threshold of <0.10, we initially identified 19 variables that are significantly associated with CLBR (Table 2; Supplementary Table S3). These 19 variables were subsequently subjected to feature selection using the Boruta algorithm, RFE, and mRMR. The Boruta analysis confirmed ten variables as important features (Figures 2A,B). Through cross-validation, the RFE analysis identified an optimal feature subset of ten variables (Figure 2C) and ranked their importance (Figure 2D). The mRMR algorithm identified ten key predictors: embryo type of transfer, cyst diameter, AMH, adenomyosis, downregulation, previous live birth history, infertility duration, P on Gn starting day, female age, and AFC. Finally, we integrated the results from all three selection methods and selected the features that were identified by all approaches. The intersection of feature sets from the three methods yielded five core variables: cyst diameter, downregulation, previous live birth history, AFC, and progesterone level on Gn starting day (Figure 2E). These core predictors were used to build a machine learning model.

**TABLE 2:** Univariate logistic regression analysis for predicting cumulative live birth.

Characteristics	B	SE	OR	CI	Z	p
Female age (years)	0.150	0.049	1.162	1.162 (1.061–1.286)	3.093	0.002
Male age (years)	0.097	0.044	1.102	1.102 (1.017–1.207)	2.230	0.026
AMH (ng/mL)	−0.217	0.088	0.805	0.805 (0.673–0.953)	−2.461	0.014
AFC	0.104	0.031	1.109	1.109 (1.047–1.182)	3.373	0.001
Duration of infertility (years)	0.137	0.070	1.147	1.147 (1.005–1.326)	1.957	0.050
Previous live birth history						
Yes	Ref	​	​	​	​	​
No	−1.050	0.479	0.350	0.35 (0.128–0.862)	−2.190	0.029
Adenomyosis						
Yes	Ref	​	​	​	​	​
No	−1.407	0.802	0.245	0.245 (0.036–0.998)	−1.755	0.079
Uterine fibroids						
Yes	Ref	​	​	​	​	​
No	−0.833	0.488	0.435	0.435 (0.157–1.092)	−1.709	0.087
Cyst diameter						
4-<6 cm	Ref	​	​	​	​	​
6-<8 cm	0.375	0.47259	1.455	1.455 (0.578–3.719)	0.793	0.428
≥8 cm	0.66	0.39642	1.934	1.934 (0.894–4.249)	1.664	0.096
COH protocol						
Ultra-long GnRH-Agonist	Ref	​	​	​	​	​
GnRH-antagonist	1.567	0.436	4.790	4.79 (2.079–11.55)	3.595	0.000
Mild stimulation	0.973	0.608	2.647	2.647 (0.816–9.146)	1.600	0.109
Long GnRH agonist	0.057	0.791	1.059	1.059 (0.198–4.874)	0.072	0.942
Natural cycle	0.973	0.962	2.647	2.647 (0.401–21.64)	1.012	0.312
PPOS	0.568	0.770	1.765	1.765 (0.374–8.357)	0.738	0.460
Downregulation						
Yes	Ref	​	​	​	​	​
No	0.970	0.357	2.638	2.638 (1.32–5.371)	2.718	0.007
LH on Gn starting day	0.208	0.110	1.231	1.231 (1.014–1.552)	1.887	0.059
P on Gn starting day	1.390	0.506	4.014	4.014 (1.65–11.99)	2.745	0.006
LH on hCG day (mIU/mL)	0.270	0.109	1.310	1.31 (1.078–1.664)	2.472	0.013
Number of large follicles on hCG day	−0.105	0.039	0.900	0.9 (0.832–0.969)	−2.719	0.007
Number of oocytes retrieved	−0.069	0.028	0.933	0.933 (0.88–0.984)	−2.438	0.015
Number of MII oocytes	−0.066	0.031	0.936	0.936 (0.878–0.993)	−2.123	0.034
Number of usable embryos	−0.091	0.047	0.913	0.913 (0.829–0.999)	−1.920	0.055
Number of good quality embryos	−0.124	0.070	0.883	0.883 (0.765–1.009)	−1.774	0.076

Results are presented as regression coefficients (B), standard errors (SE), odds ratios (OR) with 95% confidence intervals (CI), Z-statistics, and p-values. Statistical significance was set at p < 0.10. “Ref” indicates the reference category for the categorical variables. AMH, anti-Müllerian hormone; AFC, antral follicle count; COH, controlled ovarian hyperstimulation; GnRH, gonadotropin-releasing hormone; PPOS, progestin-primed ovarian stimulation; Gn, gonadotropin; LH, luteinizing hormone; P, progesterone; hCG, human chorionic gonadotropin; MII, metaphase II, oocytes.

### 3.3 Construction and evaluation of machine learning models

**FIGURE 3:**
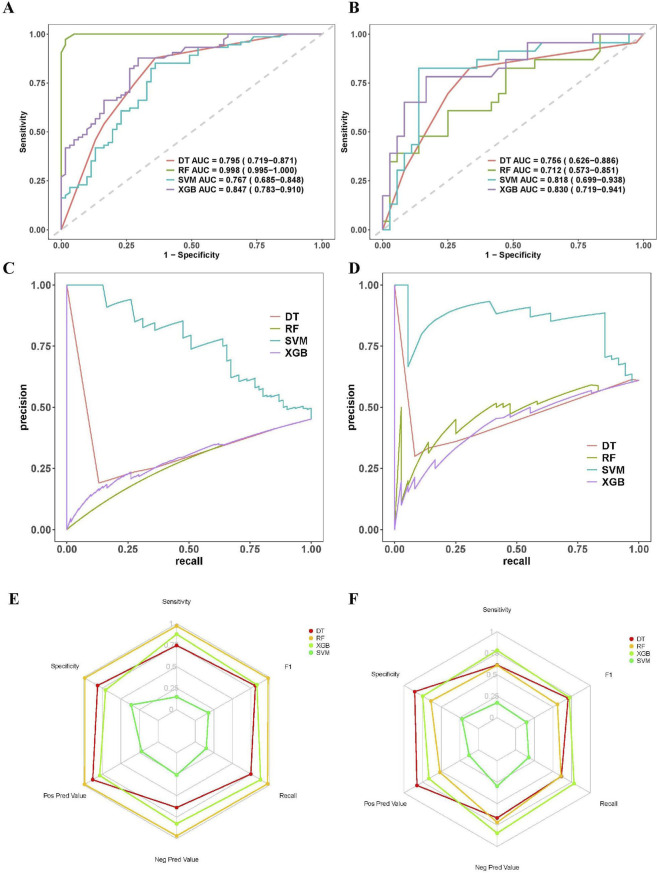
Performance comparison of the four machine learning models on training and validation sets. **(A)** ROC curves for training sets. **(B)** ROC curves for validation sets. **(C)** PR curves for training sets. **(D)** PR curves for validation sets. **(E)** Radar charts of model performance metrics Sensitivity, Specificity, Positive Predictive Value, Negative Predictive Value, Recall, and F1-score for the training sets. **(F)** Radar charts of model performance metrics Sensitivity, Specificity, Positive Predictive Value, Negative Predictive Value, Recall, and F1-score for the validation sets. Note: DT, Decision Tree; RF, Random Forest; SVM, Support Vector Machine; XGBoost, Extreme Gradient Boosting; ROC, receiver operating characteristic; AUC, area under the curve; F1, F1-score; Pos Pred Value, positive predictive value; Neg Pred Value, negative predictive value.

Based on the five core predictors selected previously, we constructed and evaluated four different machine learning models to predict CLBR in patients with OMAs after EST followed by subsequent IVF/ICSI. In the training dataset, the RF model demonstrated superior performance, with an AUC of 0.998 (95% CI: 0.995–1.000), sensitivity and specificity of 0.973 and 0.984, respectively, a balanced accuracy of 0.978, and a Brier score of only 0.0409, indicating excellent predictive ability and probability calibration. However, the performance of this model deteriorated substantially in the validation dataset, with the AUC decreasing to 0.712 (95% CI: 0.573–0.851) and the balanced accuracy dropping to 0.624, suggesting significant overfitting (Figures 3A,B,E,F; Table 3). Furthermore, the XGBoost model exhibited consistent performance across both datasets. In the validation group, XGBoost achieved the highest AUC of 0.830 (95% CI: 0.719–0.941) and a balanced accuracy of 0.766, while maintaining a good balance between sensitivity (0.783) and specificity (0.750). Furthermore, XGBoost demonstrated the best calibration performance among all models with a validation Brier score of 0.176 (95% CI: 0.145–0.233) (Figures 3A,B,E,F; Table 3). The DT model performed moderately, with a validation AUC of 0.756 (95% CI: 0.626–0.886), high specificity (0.857), but lower sensitivity (0.613) (Figures 3A,B,E,F; Table 3). The SVM model presents a unique combination of characteristics. Despite a relatively high validation AUC (0.818, 95% CI: 0.699–0.938), it had extremely low sensitivity (0.174) and specificity (0.222), resulting in a balanced accuracy of only 0.198 and a high Brier score of 0.380, indicating unreliable probability predictions (Figures 3A,B,E,F; Table 3). The Precision-Recall (PR) curves further supported this observation (Figures 3C,D). Based on comprehensive performance metrics, the XGBoost model demonstrated the best overall performance and generalization capability and was therefore selected as the final prediction model for this study.

**TABLE 3:** Comprehensive performance comparison of the four machine learning models.

Model	Dataset	AUC (95% CI)	Sensitivity	Specificity	Balanced accuracy	Brier score (95% CI)
DT	Training	0.795 (0.719–0.871)	0.747	0.812	0.78	0.171 (0.138–0.210)
	Validation	0.756 (0.626–0.886)	0.613	0.857	0.735	0.197 (0.139–0.260)
RF	Training	0.998 (0.995–1.000)	0.973	0.984	0.978	0.041 (0.032–0.055)
	Validation	0.712 (0.573–0.851)	0.609	0.639	0.624	0.219 (0.171–0.277)
XGBoost	Training	0.847 (0.783–0.910)	0.878	0.705	0.792	0.160 (0.133–0.191)
	Validation	0.830 (0.719–0.941)	0.783	0.75	0.766	0.176 (0.145–0.233)
SVM	Training	0.767 (0.685–0.848)	0.149	0.361	0.255	0.388 (0.354–0.422)
	Validation	0.818 (0.699–0.938)	0.174	0.222	0.198	0.380 (0.336–0.416)

Brier score, overall measure of calibration and accuracy (0 is perfect; lower is better); sensitivity, proportion of actual positives correctly identified; specificity, proportion of actual negatives correctly identified. AUC, area under the receiver operating characteristic curve; CI, confidence interval; DT, decision tree; RF, random forest; XGBoost, extreme gradient boosting; SVM, support vector machine.

### 3.4 Calibration and clinical utility of the models

**FIGURE 4:**
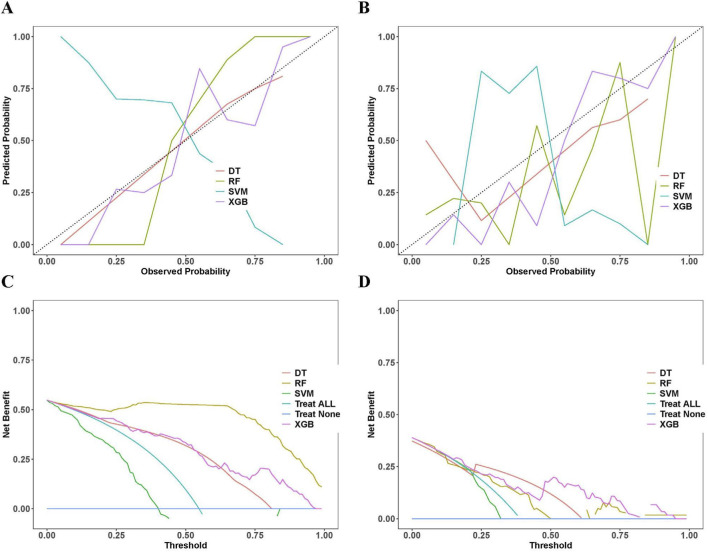
Calibration and clinical utility assessments of the four machine learning models. **(A)** Calibration curves for training sets. **(B)** Calibration curves for validation sets. **(C)** Decision curves for training sets. **(D)** Decision curves for validation sets. Note: DT, Decision Tree; RF, Random Forest; SVM, Support Vector Machine; XGBoost, Extreme Gradient Boosting; Treat All, strategy of treating all patients; Treat None, strategy of treating no patients.

To evaluate the performance of the models, we analyzed their calibration and clinical utility. The calibration curves of the XGBoost and SVM models were closer to the ideal diagonal line in both the training (Figure 4A) and validation sets (Figure 4B), indicating well-aligned predicted probabilities. In contrast, the DT and RF models showed greater deviations from the diagonal, particularly in the validation group, demonstrating poor calibration. Decision Curve Analysis was used to evaluate the clinical utility of the models by calculating the net benefit at different threshold probabilities. In both training (Figure 4C) and validation sets (Figure 4D), Decision Curve Analysis showed that between probabilities 0.1–0.8, the XGBoost and SVM models performed better than ‘Treat All’ and ‘Treat None’ strategies and other models. Using XGBoost models for clinical decisions yields greater net benefit than other models.

### 3.5 Interpretability analysis of XGBoost model

**FIGURE 5:**
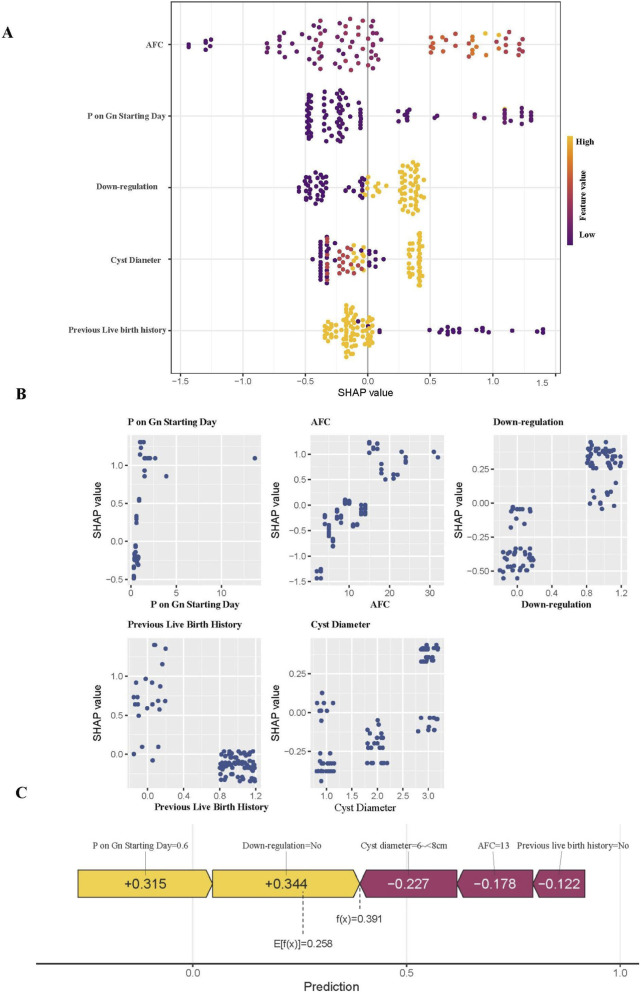
Interpretability analysis of the XGBoost model using SHAP method. **(A)** SHAP summary plot. **(B)** Dependence plots for each feature, revealing the relationship between the feature values and SHAP values. **(C)** SHAP force plot for a single case, illustrating the attribution of individualized prediction. Note: SHAP, SHapley Additive exPlanations; AFC, antral follicle count; Gn, gonadotropin; E [f(x)], expected value (base value); f(x), model prediction for individual patient.

We employed SHAP to reveal the decision-making mechanisms of the optimal XGBoost model. Based on the mean absolute SHAP values, the features were ranked in order of importance as follows: AFC, progesterone on Gn-starting day, downregulation, cyst diameter, and previous live birth history (Figure 5A). AFC emerged as the most influential predictor, with higher AFC values (orange-red dots) consistently associated with positive SHAP values (rightward distribution), indicating an increased CLBR. The feature dependence plots (Figure 5B) further reveal the specific relationship between the value of each variable and its contribution to the model’s prediction (SHAP value), intuitively visualizing the nonlinear dependency patterns. We further present the SHAP force plot for a representative case (Figure 5C). This plot quantifies how each feature pushes the prediction from the base value (the average prediction probability across all samples, E [f(x)] = 0.258) to the final predicted probability for this patient (f(x) = 0.391). For this specific case, a low progesterone level on Gn starting day (+0.315) and the absence of downregulation (+0.344) were the main drivers of the increased predicted live birth probability, while a medium-sized cyst diameter, moderate AFC, and history of previous live birth played minor negative regulatory roles. This individualized explanation provides a solid foundation for clinicians to understand and trust the model’s prediction.

## 4 Discussion

### 4.1 Summary of key findings

We developed and validated an XGBoost-based machine learning model to predict the CLBR in patients with OMAs undergoing IVF/ICSI. Cross-validation across three feature-selection algorithms identified five core predictors: AFC, progesterone level on Gn starting day, downregulation, cyst diameter, and previous live birth history. Our study provides a predictive tool with good discrimination (AUC = 0.830), calibration, and clinical benefit, while revealing model decisions through SHAP analysis for personalized clinical counseling.

### 4.2 Interpretation of key predictors

#### 4.2.1 Antral follicle count: the most important predictor of cumulative live birth

In our XGBoost model, AFC emerged as the most important predictor, and SHAP analysis indicated a significant positive contribution to the CLBR. As a core indicator for assessing the ovarian reserve, AFC directly reflects the size of the primordial follicle pool available for recruitment (La Marca et al., 2010; Tal and Seifer, 2017). Patients with higher AFC have better ovarian reserve, enabling more follicle recruitment during COH and increased oocyte retrieval, which improves chances of quality embryos and live birth (Sunkara et al., 2011; Polyzos et al., 2018). OMAs can impair the ovarian reserve through mechanisms such as local inflammation, oxidative stress, and surgical damage (Wu et al., 2021; Sanchez et al., 2017). Therefore, after undergoing EST, the baseline AFC level becomes a critical factor for assessing a patient’s remaining reproductive potential.

#### 4.2.2 Progesterone level on Gn starting day: an early warning indicator

Our model identified the progesterone level on Gn starting day as the second most important predictor, with SHAP analysis revealing that an elevated P level on the starting day negatively impacts CLBR. While the clinical focus has traditionally been on progesterone elevation during hCG administration, evidence suggests that the early follicular phase progesterone environment is equally important (Venetis et al., 2013). A slight increase in progesterone levels at initiation may indicate follicular asynchrony or premature luteinization. Bila et al. (Bila et al., 2024) noted that elevated baseline progesterone levels are associated with adverse IVF outcomes, particularly in patients with EMs. This adverse effect may occur via several mechanisms. Premature progesterone exposure can impair endometrial receptivity, causing early closure of the ‘window of implantation’ and reducing the likelihood of successful embryo implantation (Roque et al., 2019; Shi et al., 2018). Second, it may reflect abnormal granulosa cell function, which compromises the final oocyte maturation and quality. Lim et al. (Lim et al., 2024) highlighted that inappropriate progesterone levels during different phases of the ART can negatively affect pregnancy outcomes. Our results indicate that elevated progesterone levels should prompt the consideration of a freeze-all strategy to bypass endometrial factors, supporting its use as a practical monitoring marker in routine care of patients undergoing IVF.

#### 4.2.3 Downregulation: optimizing the reproductive microenvironment

An intriguing finding from our SHAP analysis was that patients who did not receive pituitary downregulation demonstrated significantly higher CLBR than those who underwent downregulation with GnRH agonist protocols (OR = 2.638, P = 0.007) (Tian et al., 2023; Cao et al., 2020). This observation appears to contradict conventional wisdom in reproductive endocrinology, as multiple meta-analyses have demonstrated that long-term pituitary downregulation with GnRH agonists can improve clinical pregnancy rates in patients with stage III-IV endometriosis undergoing IVF/ICSI (Tian et al., 2023; Cao et al., 2020). GnRH-a suppresses gonadotropin secretion, creating low levels of estrogen that inhibit ectopic lesions and reduce inflammation. Tian et al. (Tian et al., 2023) found that GnRH-a pretreatment before IVF improved clinical pregnancy and live birth rates in EMs patients. The mechanisms underlying these benefits are likely multifactorial. First, EST treatment may have already improved the pelvic environment by directly ablating endometriotic cysts, reducing local inflammatory cytokines, such as interleukin-6 and tumor necrosis factor-alpha, and eliminating the source of oxidative stress that characterizes endometriosis (Vercellini et al., 2009). Second, patients who have undergone UGES may already have a compromised ovarian reserve due to both the underlying endometriosis and the sclerotherapy procedure itself. Third, we cannot exclude the possibility of selection bias in our retrospective cohort study. However, given the retrospective nature of our study and the potential for selection bias, these findings should be validated in prospective randomized controlled trials that compare different ovarian stimulation protocols in post-EST patients.

#### 4.2.4 Cyst diameter and previous live birth history: additional prognostic factors

Cyst diameter is an independent predictor of CLBR following ART in patients who have undergone EST for OMAs. Even after EST, a larger original cyst diameter may imply more extensive damage to the ovarian tissue and a more severe local inflammatory environment, the potential harm of which may not be fully reversed by treatment (Aflatoonian, Rahmani, and Rahsepar, 2013; Cohen, et al., 2017). Wu et al. (2021) found that OMAs negatively affect outcomes primarily by affecting the quantity and quality of oocytes, with cyst size being a direct indicator of the extent of this impact. Daniilidis et al. (Kheil et al., 2022) also indirectly supported the greater threat posed by larger lesions to the ovarian reserve and reproductive outcome. Taken together, these data highlight cyst diameter as a readily quantifiable, clinically actionable metric that should inform prognostic counseling, risk stratification, and individualized ART planning after EST completion.

Previous live birth history emerged as a significant positive prognostic marker in our model, with patients who had achieved previous live births demonstrating substantially higher CLBR. This aligns with clinical evidence highlighting that previous live births are strong predictors of success (McLernon et al., 2016). In endometriosis, it signifies overcoming disease-related barriers, such as poor oocyte quality and compromised receptivity (Bonavina and Taylor, 2022). Beyond past success, it may reflect broader reproductive fitness, including embryo quality and endometrial interaction, which is not fully captured by AMH or AFC. Patients without previous live births require closer monitoring, whereas those with births can expect better outcomes. EST provides a minimally invasive option to laparoscopic cystectomy for OMAs. While cystectomy removes lesions, it inevitably damages adjacent ovarian tissue and reduces the ovarian reserve, especially in bilateral or recurrent diseases (Hamdan et al., 2015). EST, which entails ultrasound-guided aspiration followed by the instillation of absolute ethanol, promotes cyst wall sclerosis, reduces cyst size, and more effectively preserves the ovarian reserve (Aflatoonian, Rahmani, and Rahsepar, 2013; Cohen et al., 2017). Cohen et al., (2017) reported acceptable recurrence and significantly less impact on reserve than surgery. In our cohort, post-EST AFC remained favorable, supporting the success of subsequent IVF. Thus, sclerotherapy should be prioritized for fertility-seeking patients, particularly those with compromised ovarian reserves or bilateral cysts.

### 4.3 Ethanol sclerotherapy versus surgical cystectomy: a paradigm shift

The enhancement of doctor-patient communication has become more intuitive and precise, thereby significantly advancing personalized precision medicine. Artificial intelligence is paving the way for a transformative era in reproductive medicine, with applications extending to embryo morphology assessment, oocyte quality prediction, and personalized COH protocols, highlighting its substantial clinical potential (Zaninovic and Rosenwaks, 2020; Olawade et al., 2025). Our study serves as a proof of concept in this field, demonstrating that for patients with OMAs undergoing IVF following EST, ML models are not only feasible but also exceed traditional methods in terms of predictive performance and clinical utility.

### 4.4 SHAP interpretation and causal inference

While SHAP analysis significantly enhances the clinical interpretability of our machine learning model by revealing which features drive its predictions, it is crucial to emphasize that SHAP values represent statistical associations learned by the model, not biological causation. For instance, although SHAP analysis may indicate that higher progesterone levels on Gn initiation day are associated with a lower predicted likelihood of live birth in our model, this does not imply that progesterone directly causes adverse outcomes. Such associations could be influenced by underlying confounders (e.g., patients with elevated progesterone may have other unobserved characteristics affecting outcomes) or simply reflect complex patterns captured by the model from the data. Therefore, these findings should be interpreted cautiously as insights into our model’s internal decision-making logic, rather than as direct clinical causal inferences. The distinction between prediction and causation is fundamental in machine learning applications to clinical medicine. The primary value of this study lies in identifying potentially important predictors and generating testable clinical hypotheses to guide future causal research, rather than providing definitive causal evidence for immediate clinical application.

### 4.5 Subgroup analysis

To investigate the unexpected associations observed in our initial analysis (e.g., lower AFC in the live birth group), we conducted subgroup analyses. When stratified by AFC level (cutoff at 12), patients with AFC ≥12 had a significantly higher CLBR than those with AFC <12 (63.2% vs. 39.3%, P = 0.001). Similarly, when stratified by downregulation protocol, patients who underwent downregulation had a significantly higher CLBR than those who did not (65.3% vs. 35.4%, P < 0.001) (Supplementary Table S4). These findings are consistent with established clinical knowledge, suggesting that the “unexpected associations” observed in the overall dataset were likely attributable to complex interactions between subgroups, confounding factors, or selection bias in a small sample.

### 4.6 Strengths and limitations

One of the main strengths of this study is that it is among the first to develop a machine learning model for CLBR prediction, specifically in patients who have undergone EST for OMAs. We employed multiple feature selection methods (Boruta, RFE, and mRMR), ensuring that the included variables had high predictive values. We enhanced the interpretability of the model using the SHAP framework for clinical translation. The model showed good discrimination (AUC = 0.830), calibration, and clinical net benefit in the validation group, demonstrating its reliability.

However, our study has several limitations that must be acknowledged. A primary concern is the risk of overfitting, which was particularly evident in some of our initial models. For instance, the Random Forest model exhibited near-perfect performance on the training set (AUC = 0.998) but showed a substantial drop on the validation set (AUC = 0.712). This classic overfitting phenomenon highlights the risks of applying complex models to datasets with a limited sample size. It was precisely our vigilance against this risk that guided our final selection of the XGBoost model, which demonstrated the most robust and superior overall performance. Second, although we employed stratified random sampling to ensure a consistent distribution of the outcome variable, the small overall sample size (n = 194) led to chance imbalances in some baseline characteristics between the training and validation sets. This random statistical imbalance, arising from the data split, may partly explain the model’s performance drop on the validation set and could be a contributing factor to the observed overfitting. Third, our validation set is relatively small (n = 59), which can lead to wider confidence intervals for performance metrics such as the AUC and calibration curves, thereby increasing the uncertainty of our performance estimates. This implies that the AUC observed in our validation set may not be a precise representation of the model’s true performance in future patients. Fourth, our stringent feature selection strategy—requiring a variable to be selected by the intersection of three methods (Boruta, RFE, and mRMR)—creates an inherent trade-off. We acknowledge that this conservative approach, while effective in building a parsimonious and robust model, may have excluded ‘moderately informative’ predictors that did not consistently rank as top-tier across all methods. Our rationale was to prioritize model parsimony, as simpler models are typically more generalizable, less prone to overfitting, and more readily interpretable in a clinical context. Although our study included patients undergoing both cleavage-stage and blastocyst-stage embryo transfers, the limited sample size of the blastocyst group in this retrospective dataset precluded stratified analysis. Robust statistical power for such an analysis would necessitate a significantly larger, prospectively collected cohort. Collectively, these limitations—overfitting risk, chance imbalances from data splitting, and uncertainty from a small validation set—underscore the absolute necessity of external validation in larger, independent, and more diverse cohorts to confirm the model’s generalizability and clinical utility before any form of clinical application can be considered.

### 4.7 Clinical utility and personalized decision-making

The XGBoost model developed in this study has clear clinical value. It provides individualized pre-treatment CLBR predictions to support shared decision-making; a high predicted CLBR can bolster confidence and adherence, whereas a low predicted CLBR enables proactive counseling on plan modification (e.g., oocyte donation, gestational surrogacy, or other ART options) and realistic expectation setting to reduce physical, emotional, and financial burdens (Kamath et al., 2018; Cimadomo et al., 2018). Risk stratification also optimizes resource allocation by directing intensified monitoring and tailored protocols to higher-risk patients and facilitates patient stratification and evaluation of treatment efficacy. Finally, SHAP-based interpretability clarifies the key determinants of IVF outcomes in patients with OMAs, guiding the development of more precise management strategies.

## 5 Conclusion

This study developed a high-performing XGBoost model with SHAP interpretability to predict CLBR in OMA patients following ultrasound-guided EST. The model demonstrated good discrimination (AUC = 0.830–0.847), good calibration, and superior clinical utility compared to traditional logistic regression. Importantly, SHAP analysis provided transparent, individualized interpretation of the model’s predictions, identifying AFC, progesterone on Gn starting day, use of downregulation, cyst diameter, and previous live birth history as the five most influential predictors. This interpretable AI approach not only enhances model trustworthiness but also reveals complex, non-linear relationships between predictors and outcomes that are difficult to capture with conventional statistical methods. Clinically, our model serves as a valuable decision-support tool for personalized patient counseling, enabling clinicians to provide evidence-based, individualized prognostic information and optimize treatment strategies for this challenging patient population. Prospective validation is warranted before widespread clinical implementation.

## Data Availability

The original contributions presented in the study are included in the article/Supplementary Material, further inquiries can be directed to the corresponding authors.

## Glossary

AFC: Antral Follicle Count
AI: Artificial Intelligence
AMH: Anti-Müllerian Hormone
ART: Assisted Reproductive Technology
AUC-ROC: Area Under the Receiver Operating Characteristic Curve
BMI: Body Mass Index
CI: Confidence Interval
CLBR: Cumulative Live Birth Rate
COH: Controlled Ovarian Hyperstimulation
DT: Decision Tree
EMs: Endometriosis
EMRS: Electronic Medical Record System
EPV: Events Per Variables
EST: Ethanol Sclerotherapy
FET: Freeze-Thaw Embryo Transfer
GnRH: Gonadotropin-Releasing Hormone
hCG: Human Chorionic Gonadotropin
ICC: Intraclass Correlation Coefficient
ICSI: Intracytoplasmic Sperm Injection
IQR: Interquartile Range
IRR: Inter-rater reliability
IVF: *In Vitro* Fertilization
MAR: Missing At Random
MICE: Multiple Imputation by Chained Equations
MII: Metaphase II (mature oocytes)
ML: Machine Learning
mRMR: Maximum Relevance and Minimum Redundancy
NPV: negative predictive value
OMA: Ovarian Endometrioma
PGT-A: Preimplantation genetic testing for aneuploidy
PPV: positive predictive value
PR: Precision-Recall
RF: Random Forest
RFE: Recursive Feature Elimination
SHAP: SHapley Additive exPlanations
SSAML: Sample Size Analysis for Machine Learning
SVM: Support Vector Machine
VIF: Variance Inflation Factor
TRIPOD: Transparent Reporting of a multivariable prediction model for Individual Prognosis or Diagnosis
XGBoost: Extreme Gradient Boosting
