# Supplementary material for: Identifying key determinants of cumulative live birth in women with ovarian endometrioma undergoing ethanol sclerotherapy followed by in vitro fertilization or intracytoplasmic sperm injection: an interpretable machine learning analysis
Source: Front Cell Dev Biol. 2026 Mar 26;14:1742816. doi: 10.3389/fcell.2026.1742816 (PMC13062204; doi:10.3389/fcell.2026.1742816)
Supplement: Supplementary file 3 [file Table1.docx]

| Variables | VIF |
| --- | --- |
| Pituary Downregulation | 1.26 |
| Progesterone on Gn initiation day | 1.18 |
| Cyst diameter (mm) | 1.11 |
| AFC | 1.08 |
| Previous live birth history | 1.03 |

**Supplementary Table S1. Variance Inflation Factor Analysis for Multicollinearity Assessment.**

Note: This table presents the VIF values for the five variables selected through feature selection and included in the final predictive model. These low VIF values confirm the absence of significant multicollinearity among the selected features, supporting the stability and reliability of the model coefficients. AFC, antral follicle count; Gn, gonadotropin; VIF, variance inflation factor.
