# Supplementary material for: Identifying key determinants of cumulative live birth in women with ovarian endometrioma undergoing ethanol sclerotherapy followed by in vitro fertilization or intracytoplasmic sperm injection: an interpretable machine learning analysis
Source: Front Cell Dev Biol. 2026 Mar 26;14:1742816. doi: 10.3389/fcell.2026.1742816 (PMC13062204; doi:10.3389/fcell.2026.1742816)
Supplement: Supplementary file 4 [file Table5.docx]

Supplementary Material

# Supplementary Tables

**Supplementary Table S1. Variance Inflation Factor Analysis for Multicollinearity Assessment.**

This table presents the VIF values for the five variables selected through feature selection and included in the final predictive model. These low VIF values confirm the absence of significant multicollinearity among the selected features, supporting the stability and reliability of the model coefficients.

Note: AFC, antral follicle count; Gn, gonadotropin; VIF, variance inflation factor.

**Supplementary TableS2: Additional Baseline Characteristics of Study Population.**

Supplementary Table S2 presents comprehensive baseline characteristics of the study population, comparing participants who achieved cumulative live birth versus those who did not, as well as training and validation set distributions. Data are presented as mean ± standard deviation for normally distributed continuous variables, median [interquartile range] for non-normally distributed continuous variables, and frequency (percentage) for categorical variables.

Note: p-value for comparison between live birth and non-live birth groups using independent t-test for normally distributed continuous variables, Mann-Whitney U test for non-normally distributed continuous variables, and chi-square test or Fisher's exact test for categorical variables. p^*^ compares the live‑birth vs no‑live‑birth groups; p^#^ compares the training vs validation sets. IVF, in vitro fertilization; ICSI, intracytoplasmic sperm injection; GnRH, gonadotropin-releasing hormone; PPOS, progestin-primed ovarian stimulation; bE_2_, baseline estradiol; bFSH, baseline follicle-stimulating hormone; bLH, baseline luteinizing hormone; bP, baseline progesterone; hCG, human chorionic gonadotropin; MII, metaphase II oocytes.

**Supplementary Table S3: Additional Univariate Logistic Regression Analysis for Cumulative Live Birth Prediction.**

Supplementary Table S3 presents the results of univariate logistic regression analysis examining the association between additional baseline characteristics and cumulative live birth outcomes. Each variable was analyzed individually to assess its predictive value for cumulative live birth rate following ethanol sclerotherapy and subsequent IVF/ICSI treatment.

Note: Results are presented as regression coefficient (B), standard error (SE), odds ratio (OR) with 95% confidence interval (CI), Z-statistic, and p-value. Variables with p < 0.10 in univariate analysis were considered for inclusion in the multivariate feature selection process. For categorical variables, the reference category is indicated as "Ref." bFSH, baseline follicle-stimulating hormone; bLH, baseline luteinizing hormone; bP, baseline progesterone; bPRL, baseline prolactin; bE_2_, baseline estradiol; bT, baseline testosterone; BMI, body mass index; IVF, in vitro fertilization; ICSI, intracytoplasmic sperm injection; hCG, human chorionic gonadotropin; E2, estradiol; FSH, follicle-stimulating hormone; LH, luteinizing hormone.

**Supplementary Table S4. Subgroup Analysis of Cumulative Live Birth Rate.**

Subgroup analysis of CLBR stratified by AFC and downregulation. Data are presented as n/N (%), representing the number of patients with a live birth out of the total number in the subgroup. P-values were calculated using the Chi-square test or Fisher's exact test.

Note: AFC, Antral Follicle Count; CLBR, Cumulative Live Birth Rate.
