# Supplementary material for: Identifying key determinants of cumulative live birth in women with ovarian endometrioma undergoing ethanol sclerotherapy followed by in vitro fertilization or intracytoplasmic sperm injection: an interpretable machine learning analysis
Source: Front Cell Dev Biol. 2026 Mar 26;14:1742816. doi: 10.3389/fcell.2026.1742816 (PMC13062204; doi:10.3389/fcell.2026.1742816)
Supplement: Supplementary file 5 [file Table4.docx]

| Subgroup Analysis | CLBR (n/N) | CLBR (%) | P-value |
| --- | --- | --- | --- |
| By AFC |  |  |  |
| AFC ≥12 | 55/87 | 63.2% |  |
| AFC <12 | 42/107 | 39.3% |  |
| P-value |  |  | 0.001 |
|  |  |  |  |
| By Downregulation |  |  |  |
| With downregulation | 62/95 | 65.3% |  |
| Without downregulation | 35/99 | 35.4% |  |
| P-value |  |  | ＜0.001 |

Supplementary Table S4. Subgroup Analysis of Cumulative Live Birth Rate.

Note: Data are presented as n/N (%), representing the number of patients with a cumulative live birth out of the total number in the subgroup. P-values were calculated using the Chi-square test or Fisher's exact test. AFC, Antral Follicle Count; CLBR, Cumulative Live Birth Rate.
