# Supplementary material for: Identifying key determinants of cumulative live birth in women with ovarian endometrioma undergoing ethanol sclerotherapy followed by in vitro fertilization or intracytoplasmic sperm injection: an interpretable machine learning analysis
Source: Front Cell Dev Biol. 2026 Mar 26;14:1742816. doi: 10.3389/fcell.2026.1742816 (PMC13062204; doi:10.3389/fcell.2026.1742816)
Supplement: Supplementary file 6 [file DataSheet1.docx]

Supplementary Material

## Supplementary Figures

##
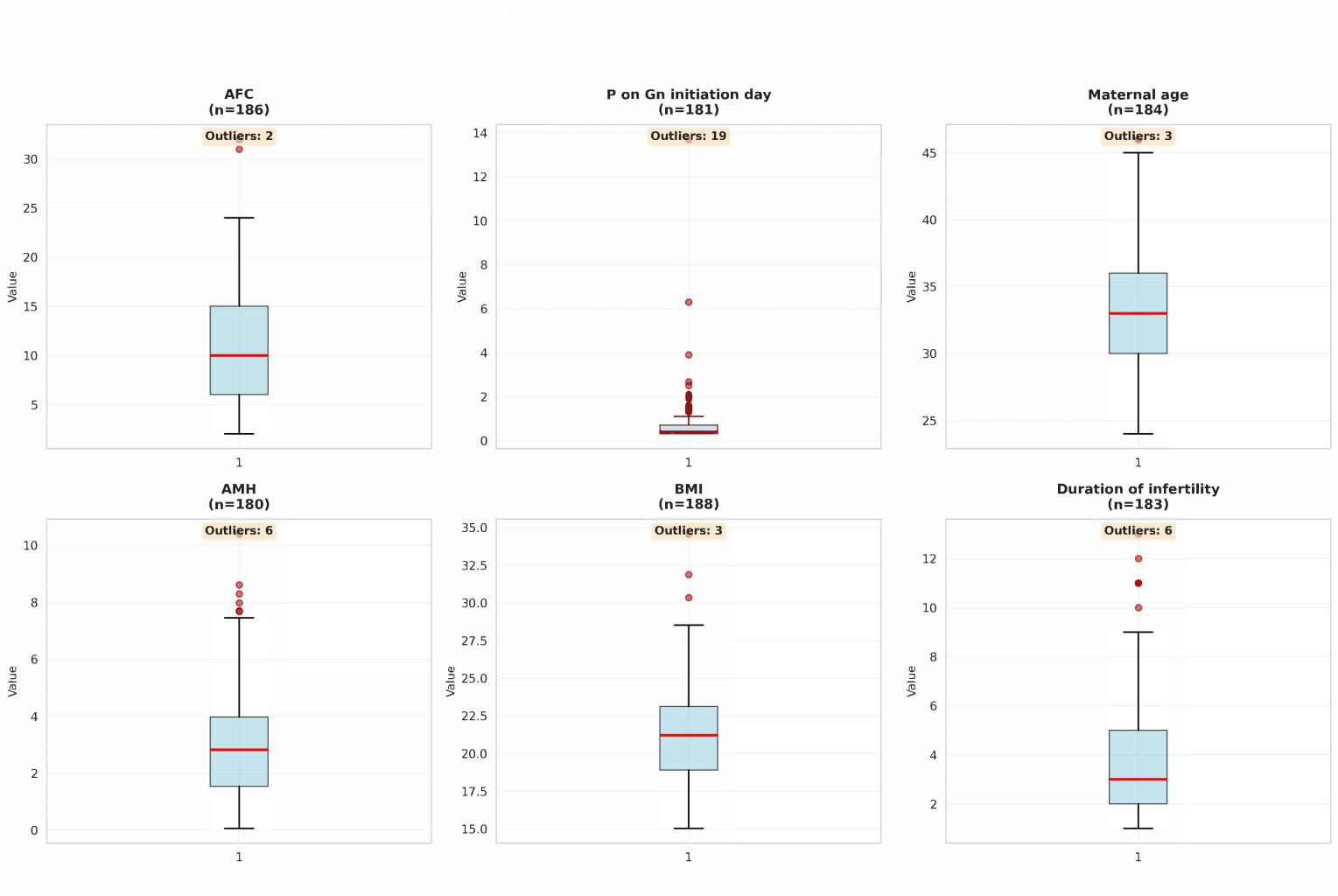


**Supplementary Figure S1. Boxplots for Outlier Detection in Continuous Variables.** Note: The box represents the IQR (25th to 75th percentile), with the red horizontal line indicating the median. Whiskers extend to 1.5*IQR from the box edges. Data points beyond the whiskers are identified as outliers using the standard 1.5*IQR criterion(Q1-1.5*IQR or above Q3+1.5*IQR) and are displayed as individual red circles. AFC, antral follicle count; AMH, anti-Müllerian hormone; BMI, body mass index; P, Progesterone; Gn, gonadotropin; IQR, interquartile range.


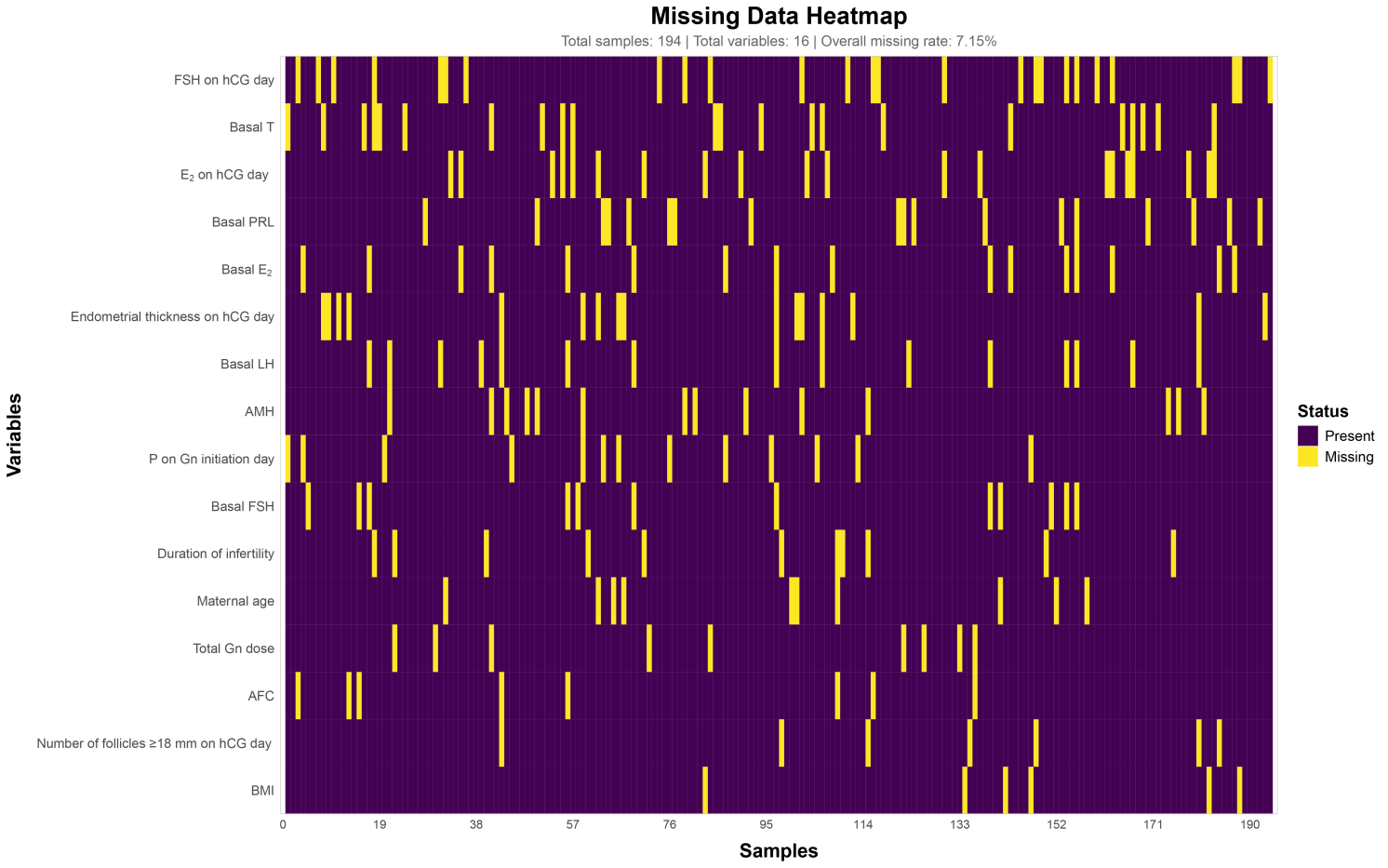


**Supplementary Figure S2. Heatmap Visualization of Missing Data Pattern.** Note: Each cell represents a single data point, with purple indicating present data and yellow indicating missing data. The overall missing rate across the entire dataset was 7.15%. Visual inspection of the heatmap reveals a scattered, non-systematic distribution of missing values across samples and variables. Purple cells indicate present data; yellow cells indicate missing data. The x-axis represents individual samples (n=194), and the y-axis represents variables (n=16). AFC, antral follicle count; AMH, anti-Müllerian hormone; BMI, body mass index; E₂, estradiol; FSH, follicle-stimulating hormone; Gn, gonadotropin; hCG, human chorionic gonadotropin; LH, luteinizing hormone; P, progesterone; PRL, prolactin; T, testosterone.


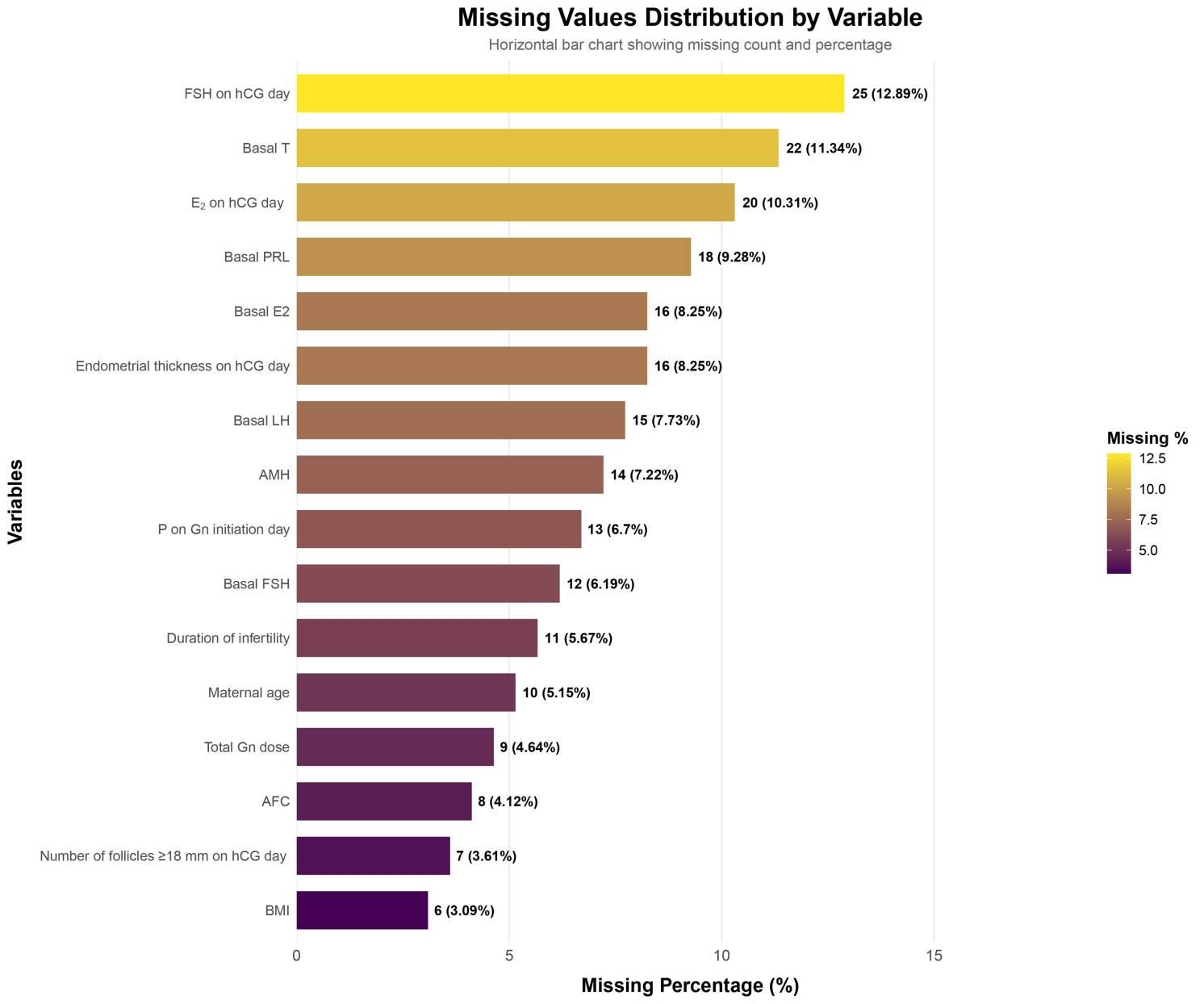


**Supplementary Figure S3. Distribution of Missing Values Across Variables.** Note: Bar chart showing the percentage of missing values for each of the 16 variables. AFC, antral follicle count; AMH, anti-Müllerian hormone; BMI, body mass index; E₂, estradiol; FSH, follicle-stimulating hormone; Gn, gonadotropin; hCG, human chorionic gonadotropin; LH, luteinizing hormone; P, progesterone; PRL, prolactin; T, testosterone.
